# Teardrop Risk Field-Based Spatiotemporal Path Planning Method for Mobile Robots in Dynamic Environments

**DOI:** 10.3390/s26154681

**Published:** 2026-07-23

**Authors:** Youkun Li, Guodong Xu, Xiaolong Zhang, Yueping Wang, Deqi Zuo

**Affiliations:** School of Mechanical and Transportation Engineering, Southwest Forestry University, Kunming 650224, China

**Keywords:** spatiotemporal joint planning, RRT algorithm, dynamic collision avoidance, teardrop risk field

## Abstract

Navigating dynamic environments requires autonomous robots to balance computational efficiency with safety. Traditional isotropic risk fields ignore obstacle velocities, causing overly conservative lateral avoidance or insufficient margins against head-on collisions. To address this, we propose TRF-ST-RRT, a spatiotemporal RRT framework driven by our core innovation: a velocity-based “teardrop” risk field (TRF). By constructing an anisotropic safety corridor, the TRF dynamically extends its leading edge based on kinematic constraints. This explicitly forces the planner to navigate behind or alongside dynamic obstacles, ensuring robust spatial buffers against oncoming threats. To efficiently deploy this field, a supporting three-layer probabilistic hybrid sampling strategy is integrated to accelerate the real-time 3D spatiotemporal search. Finally, line-of-sight path simplification and subsequent temporal reconstruction, combined with segmented Bézier smoothing, are applied to guarantee trajectory executability. Extensive ablation studies and ROS2 simulations demonstrate that, compared to individual state-of-the-art baselines such as ORCA, MPPI, and ST-RRT*, TRF-ST-RRT substantially reduces planning time and iteration counts while delivering notable improvements in both overall driving efficiency and dynamic evasion success rates.

## 1. Introduction

Path planning, as a core component of the navigation system for autonomous mobile robots, is primarily responsible for planning a path that balances safety, dynamic smoothness and execution efficiency, based on global maps and real-time sensor localization data [1,2]. Early global planning algorithms were unable to cope with sudden changes in dynamic environments, while local planning algorithms were highly prone to ‘local minima’ and ‘controller oscillations’ [3,4] in the presence of multiple dynamic obstacles, rendering them incapable of planning globally optimal paths [5]. Path planning in dynamic environments has become a hot topic of research in the fields of robotics and autonomous driving [6]. Real-time adaptability and robustness against perception uncertainties and sensor noise are the most critical determinants of success in these dense dynamic interactions [7]. For complex navigation tasks in dynamic environments, spatiotemporal trajectory planning achieves coupled optimization of path and time by introducing the temporal dimension. Such methods are further categorized into spatiotemporal decoupled planning and spatiotemporal joint planning. The former first plans a geometric path and then assigns a velocity profile; while it has lower computational complexity, it is prone to local deadlocks in scenarios involving multiple dynamic obstacles [8]. The latter searches directly in (x, y, t) space, simultaneously optimizing spatial position and passage time, and thus demonstrates greater adaptability in highly dynamic environments. The spatiotemporal joint planning architecture directly employs a synchronized spatiotemporal approach, achieving mutual coupling between spatial geometry and time, thereby enabling the robot to traverse specific spaces at specific times and precisely address interaction conflicts [9]. However, as the temporal dimension increases, the search space grows exponentially, and the system faces computational delays and bottlenecks.

In navigation tasks involving dynamic maps, early local planning algorithms were widely adopted due to their real-time capabilities [10,11]. For example, the Optimal Reciprocal Collision Avoidance (ORCA) algorithm [12], which emerged shortly after the classic Velocity Obstacle (VO) method [13], enabled rapid obstacle avoidance by multiple agents in velocity space. However, traditional ORCA relies heavily on perfect sensing and strict reciprocal assumptions, which can easily lead to deadlocks in crowded spaces; to address this, Tamás Füzesi et al. proposed an enhanced ORCA framework that relaxes these limitations by incorporating reinforcement learning, successfully improving navigation success rates in dense crowds [14]. In contrast, the Model Predictive Path Integral (MPPI) [15,16] algorithm, which has gained popularity in recent years, handles complex local dynamical systems through extensive random sampling of the model. To mitigate the jerky control inputs often generated by MPPI’s high-frequency sampling noise, Takato Miura et al. introduced a spline-interpolated model predictive path integral control with Stein variational inference, effectively ensuring kinematic smoothness while safely avoiding obstacles [17]. Furthermore, the reliable execution of these planned collision-free paths fundamentally relies on highly robust underlying tracking control. To this end, N.-H. Tran et al. proposed [18] a remarkably innovative and comprehensive control framework that seamlessly integrates the generation of RRT reference trajectories with a highly robust trajectory tracking controller. By incorporating a Radial Basis Function (RBF) neural network to compensate for unmodeled dynamics and utilizing Particle Swarm Optimization (PSO) for rigorous parameter tuning, their Integrated Sliding Mode Control (SMC) architecture achieved outstanding tracking precision, exceptional stability, and superior resilience against severe external disturbances and model uncertainties.

In recent years, spatiotemporal joint planning algorithms have achieved breakthroughs in many fields; Francesco Grothe [19] et al. proposed the Spatiotemporal Rapidly exploring Random Tree Star (ST-RRT*) algorithm for complex dynamic environments, successfully introducing an asymptotic optimization framework into spatiotemporal joint planning. To ensure sufficient safety, Jianhua Guo [20] et al. introduced mixed-integer variables to represent the boundaries of dynamic obstacles, aiming for global optimization; however, the computational cost increased exponentially. Haonan Hu [21] et al. proposed a spatiotemporally variable-step A* algorithm combined with B-spline smoothing for wheeled mobile robots, which effectively reduced computation time and ensured smooth navigation. The effectiveness of incorporating the temporal dimension has been further validated by W. Burzyński and W. Stecz, who proposed a Multiplatform Spacetime RRT approach that constructs explicit “spacetime tunnels” to efficiently and safely route swarms of drones through highly dynamic multi-obstacle scenarios [22].

Traditional obstacle avoidance planning algorithms remain short-sighted approaches lacking long-range prediction; in game scenarios with a high density of dynamic obstacles, they are highly prone to becoming trapped in local optima or leading to deadlocks. Furthermore, in environments with a high density of dynamic obstacles, the MPPI algorithm often requires exponential sampling to avoid breaching safety boundaries, resulting in a sharp increase in computational load and causing significant real-time delays; Among spatiotemporal joint planning methods, the high-frequency parent node reconnection mechanism in ST-RRT* still faces computational bottlenecks in complex dynamic game scenarios; failure to reserve sufficient safety margins may also lead to collision risks, and the replanning process is highly prone to state transitions and termination. To address these shortcomings, constructing unified risk fields to explicitly guide sampling-based planners has emerged as a highly effective solution. For instance, Ren Xin et al. proposed an innovative RiskMap that encodes dynamic context uncertainties into an explicit differentiable risk field, effectively simplifying downstream planning and significantly improving driving safety [23]. Inspired by this paradigm, establishing an advanced anisotropic risk field to directly manipulate the spatiotemporal search space and secure sufficient collision buffers is highly necessary.

Specifically, this paper proposes the TRF-ST-RRT framework. Our main contributions are summarized as follows:

(1) Velocity-Vector-Based Teardrop Risk Field (TRF) and Real-Time Computational Support: The core innovation is the velocity-dependent TRF model, which constructs anisotropic safety corridors to rectify the inherent flaws of traditional isotropic Gaussian fields, such as the conflict between lateral conservatism and insufficient frontal safety margins. By explicitly modeling obstacle kinematics, the TRF forces the planner to secure proactive collision-free maneuvers behind or alongside moving threats. To facilitate the real-time deployment of this field within high-dimensional spatiotemporal searches, we integrated a three-layer probabilistic hybrid sampling strategy. By synergizing goal-biased and A*-heuristic-guided exploration with uniform random sampling, this mechanism effectively alleviates the computational bottlenecks of high-dimensional exploration while rigorously preserving the framework’s probabilistic completeness.
(2) Trajectory Post-Processing and Kinematic Refinement: A specialized post-processing framework was developed to bridge the gap between abstract path generation and physical execution. Through the integration of Line-of-Sight (LOS) temporal reallocation and segmented Bézier curve smoothing [24,25], this module successfully bridges planning optimality and motion executability. This synergy significantly enhances both navigational efficiency and kinematic smoothness, ensuring that the generated trajectories are robustly executable under strict dynamic constraints.

## 2. Spatiotemporal Coarse Path Generation

The joint spatiotemporal planning algorithm operates in two core phases. First, it constructs a spatiotemporal grid map using 3D spatiotemporal tubes to characterize obstacle kinematics, delineating navigable regions and safe time windows over a predictive horizon. Second, leveraging this representation, an optimized RRT searches the 3D state space (x, y, t) to yield a coarse spatiotemporal path that proactively bypasses dynamic obstacles.

### 2.1. Construction of Spatiotemporal Gridded Maps

Traditional 2D (x, y) maps fail to capture the temporal evolution of dynamic obstacles, constraining the planner’s predictive capabilities. To enable joint spatiotemporal searches, we construct a 3D state space (x, y, t), where the temporal axis t maps obstacle trajectories into spatiotemporal occupancy volumes. Selecting the time discretization step Δ*t* necessitates a strict trade-off: an excessively large step risks missed collisions, whereas a minute step inflates the computational burden [26]. To optimize this balance in practice, we draw inspiration from the Nyquist sampling theorem to formulate the discrete time step as follows:

(1) $$∆t_{opt}=\alpha \times \frac{R_{robot}+R_{min\_obs}+D_{margin}}{V_{max\_obs}+V_{max\_robot}}$$

where α is the discrete-time safety factor, ranging from 0.3 to 0.5. Setting this factor below 0.3 generates redundant spatiotemporal search layers that exponentially inflate computation time without yielding tangible safety benefits. Exceeding the 0.5 threshold excessively widens time intervals, violating continuous collision detection limits and triggering severe risks of inter-step collision traversal, commonly referred to as the tunneling effect.

**Figure 1:**
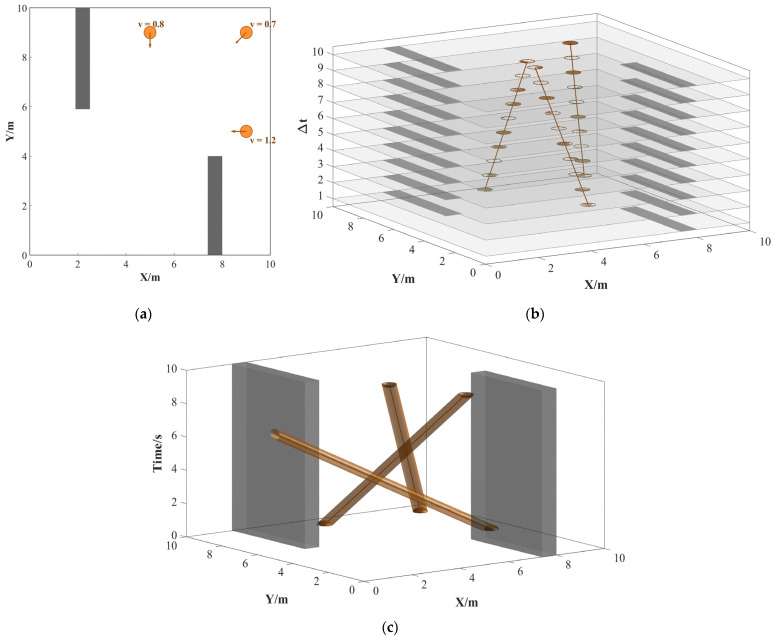
Visualization of the environmental map: (**a**) 2D map containing dynamic obstacles and static obstacles; (**b**) Schematic diagram of a discrete-time local region; (**c**) 3D spatiotemporal pipe diagram generated from dynamic and static maps.

In a 3D spatiotemporal grid map, the inclination of an obstacle within the spatiotemporal space is intrinsically determined by its velocity. Static obstacles extend vertically upward along the time axis, whereas dynamic obstacles extend at an angle, with their degree of inclination relative to the time axis being positively correlated with their velocities. Figure 1 illustrates this spatiotemporal relationship in detail. Figure 1a depicts the initial two-dimensional spatial layout, where gray regions represent static obstacles and orange circles represent dynamic obstacles, each characterized by a distinct initial position, velocity, and heading direction. Figure 1b demonstrates the evolution of obstacle positions across discrete time steps. Over time, the spatial coordinates of static obstacles remain strictly constant, while the positions of dynamic obstacles update distinctly at each discrete time layer based on their current kinematic states. Figure 1c visualizes the continuous spatiotemporal volume, which establishes a direct discrete-to-continuous corresponding relationship with Figure 1b by acting as the macroscopic representation of countless discrete time slices densely accumulated along the time axis, and within this continuous space, the two-dimensional footprints of static obstacles are directly extruded into vertical prisms, whereas the moving circular dynamic obstacles evolve into tilted cylindrical tubes.

### 2.2. Three-Layer Probabilistic Mixed Spatiotemporal RRT Algorithm

The Rapidly exploring Random Tree (RRT) is a classic sampling-based path planner [27,28]. Rooted at the start configuration, it iteratively samples the state space, extending a fixed step from the nearest tree node toward each random sample. Collision-free extensions continuously append new nodes until reaching the target region, where the trajectory is extracted via backtracking. While highly efficient at discovering feasible routes in high-dimensional complex spaces, RRT’s inherent randomness typically yields tortuous, suboptimal paths rather than globally shortest solutions.

Integrating the temporal dimension into joint spatiotemporal planning exponentially expands the search space. Since the node-rewiring mechanism of RRT* [29] incurs prohibitive computational overhead during high-dimensional spatiotemporal searches, this paper adopts the standard RRT architecture as its backbone. To accelerate convergence, we augment this framework with heuristic-guided exploration and a goal-biased sampling mechanism.

#### 2.2.1. Goal-Biased Sampling

Conventional RRT employs uniform random sampling across the entire state space, inherently suffering from sluggish goal convergence. To mitigate this, goal-biased sampling explicitly designates the target configuration as the sampled node with a predefined probability, actively steering the search tree toward the goal region. Given a goal-bias probability $P_{goal}\in [0,\;1]$, during each expansion step, the algorithm dictates whether to select the goal directly or to generate a sample centered within the spatiotemporal space, proceeding as follows:

(2) $$X_{rand}=\left\{\begin{array}{c} X_{goal},\;\;\;\;\;p=P_{goal}\\ Uniform\left(X_{free}\right),\;p=(1-P_{goal}) \end{array}\right.$$

This straightforward yet highly efficient probabilistic biasing mechanism has been demonstrated to accelerate planning while strictly preserving the probabilistic completeness of the RRT algorithm [30].

#### 2.2.2. Heuristic Guided Search

While goal-biased sampling performs excellently in open environments, it still suffers from excessive iteration overhead when traversing narrow passages within complex scenarios. To address this limitation, we integrate a heuristic-guided search inspired by the A* algorithm. This mechanism enables the RRT to sample configurations, with a predefined probability, along a baseline guiding path planned on a 2D static map that temporarily ignores dynamic obstacles [31]. The heuristic function is formulated using the Chebyshev distance:

(3) $$h\left(n\right)=\max\left(\left|n_{x}-g_{x}\right|,\;\left|n_{y}-g_{y}\right|\right)$$

where ($n_{x},\;n_{y}$) denotes the pixel index coordinates of the current node, and ($g_{x},g_{y}$) represents the pixel coordinates of the target. Assuming a uniform movement cost of 1, g(n) signifies the accumulated step count from the start configuration to the current node. Consequently, the A* evaluation function is formulated as follows:

(4) $$f\left(n\right)=g\left(n\right)+h\left(n\right)$$

Upon activating the A* heuristic bias, a node $P_{guide}[k]$ is randomly selected from the guiding path and augmented with a Gaussian perturbation. Consequently, the generation of the final sampled configuration is governed by a three-layer probabilistic hybrid model [32]:

(5) $$X_{rand}=\left\{\begin{array}{c} X_{goal},\;\;\;\;\;\;\;\;\;p=P_{goal}\\ P_{guide}\left[k\right]+\epsilon ,\epsilon ∼N\left(0,\sigma^{2}I\right),\;p=\left(1-P_{goal}\right)P_{A^{*}}\\ \;\;\;Uniform\left(X_{free}\right),\;\;\;\;\;p=\left(1-P_{goal}\right)\left(1-P_{A^{*}}\right) \end{array}\right.$$

The A*-guided path biases exploration toward known feasible regions, thereby mitigating redundant RRT sampling within narrow passages. Concurrently, the Gaussian perturbation distributes sampled configurations within the neighborhood of the guiding path; this preserves the capability to explore the globally optimal path while preventing planning failures caused by the temporary occupation of the path centerline by dynamic obstacles.

This ultimately resulted in a three-layer probabilistic mixed sampling algorithm, as described in Algorithm 1.
**Algorithm 1:** Three-Layer Hybrid SamplingInput: x_goal, Path_guide, p_goal = 0.25, p_astar = 0.40, σ = 0.3 Output: Sample point x_rand 1: rand ← Random(0, 1) 2: if rand < p_goal then 3:        x_rand ← x_goal 4: else if rand < p_goal + p_astar and |Path_guide| > 0 then 5:        idx ← RandomInt(0, |Path_guide| − 1) 6:        p_guide ← Path_guide[idx] 7:        noise_x ← Gaussian(0, σ) 8:        noise_y ← Gaussian(0, σ) 9:        x_rand ← (p_guide.x + noise_x, p_guide.y + noise_y) 10: else 11:        x_rand.x ← Uniform(x_min, x_max) 12:        x_rand.y ← Uniform(y_min, y_max) 13: end if 14: return x_rand

## 3. Construction of a Teardrop Risk Field and Post-Processing of Trajectories

Conventional Gaussian-based risk fields treat dynamic obstacles as static; their isotropic boundaries cause excessive lateral conservatism but insufficient frontal safety margins during head-on encounters, particularly in multi-agent settings. To resolve these limitations, this paper proposes a velocity-vector-based TRF that constructs anisotropic safety corridors by explicitly modeling obstacle kinematics. This mechanism scales frontal elongation proportionally to the obstacle’s velocity, securing adequate frontal clearance for safe braking while minimizing lateral margins.

### 3.1. Geometry and Construction of the Teardrop Risk Field

At low obstacle velocities, a threshold is introduced below which the risk field degrades into a conventional circular Gaussian field, omitting frontal elongation to minimize redundant overhead. This degradation is justified because low-speed trajectory prediction errors are negligible, rendering frontal elongation computationally futile with marginal engineering significance.

In this isotropic state, the field propagates uniformly in all directions. To conserve computational resources, the risk value is truncated to zero beyond the baseline radius. Accordingly, the minimum safety radius is defined as follows:

(6) $$r_{base}=r_{0}+r_{static}$$

where $r_{base}$ denotes the baseline safety radius, $r_{0}$ represents the inherent radius of the dynamic obstacle, and $r_{static}$ defines the inflated safety margin.

When an obstacle’s velocity falls below the predefined threshold, it can be modeled as a quasi-static entity. In this scenario, the collision risk depends primarily on spatial distance, exhibiting an isotropic circular profile. The risk field function under sub-threshold velocities is defined as:

(7) $$r_{1}=\left\{\begin{array}{c} e^{-\lambda {\left(\frac{d}{r_{base}}\right)}^{2}},\;\;d\leq r_{base}\\ \;\;0,\;\;d>r_{base} \end{array}\right.$$

where *d* denotes the Euclidean distance from the current spatial node to the center of the dynamic obstacle, and λ represents the risk decay coefficient that modulates the steepness of the risk attenuation relative to distance.

To quantify the orientation alignment of a node relative to the exact heading of the dynamic obstacle, the heading factor f is defined as follows:

(8) $$f=\frac{1+\cos\theta }{2}$$

This factor smoothly maps the node’s frontal collision risk onto the [0, 1] interval:

When θ = 0°, the node aligns directly with the obstacle’s heading, indicating extreme risk.

When θ = ±90°, the node lies laterally to the heading, representing moderate risk.

When θ = 180°, the node is positioned directly behind the obstacle, signifying the minimum risk.

Moreover, to prevent the frontal risk field from expanding unboundedly under high-speed conditions—which could entirely blockade the navigable passage and yield an unsolvable planning state—an upper stretch threshold is strictly enforced.

(9) $$L_{front}=\min\left(\frac{v_{eff}^{2}}{2a_{max}},L_{max}\right)$$

where $v_{eff}$ denotes the velocity exceeding the predefined threshold, $L_{front}$ represents the field elongation, $L_{max}$ signifies the maximum elongation threshold, and $a_{max}$ is the maximum braking deceleration.

The normalized distance $e_{d}$ is computed distinctively depending on whether the node resides ahead of or behind the obstacle:

(10) $$e_{d}=\sqrt{{\left(\frac{f_{c}}{r_{front}}\right)}^{2}+{\left(\frac{s_{c}}{r_{lat}}\right)}^{2}}$$

where $f_{c}$ denotes the longitudinal projection distance of the spatial node along the obstacle’s forward velocity vector; $s_{c}$ represents the lateral projection distance orthogonal to the obstacle’s heading; $r_{front}$ and $r_{lat}$ signify the frontal and lateral risk radii of the dynamic obstacle, respectively.

To further accentuate the risk weight along the axis of motion, a directional gain function is incorporated:

(11) $$g\left(f\right)=1+0.5f$$

Ultimately, the overall risk value associated with the dynamic obstacle is formulated as follows:

(12) $$r_{2}=\left\{\begin{array}{c} \min\left(1,g\left(f\right)\times e^{-\lambda e_{d}^{2}}\right),\;e_{d}\leq 1\\ 0,\;\;\;\;\;\;\;\;\;e_{d}>1 \end{array}\right.$$

This ultimately resulted in a TRF model, as shown in Algorithm 2:
**Algorithm 2:** Teardrop Risk Field ConstructionInput: Obstacle obs, query point p, time t Output: Risk value Risk(p, t) Parameters: v_threshold = 0.25 m/s, a_max = 0.8 m/s^2^, L_max = 1.50 m, λ = 0.3, γ = 1.5, r_base = 0.45 m 1: p_pred ← obs.start_pos + obs.velocity × t 2: Δp ← p − p_pred 3: d ← ||Δp|| 4: v ← ||obs.velocity|| 5: if v < v_threshold then 6:        if d < r_base then 7:          return exp(−d/λ) 8:        end if 9: else 10:        v_dir ← obs.velocity/v 11:        f ← (Δp · v_dir)/d 12:        L_front ← min(v^2^/(2 × a_max), L_max) 13:        r_eff ← r_base + L_front × max(1 − f, 0) 14:        if d < r_eff then 15:          return exp(−d/λ) × (1 + γ × max(f, 0)) 16:        end if 17: end if 18: return 0

### 3.2. Effectiveness of the Teardrop Risk Field

**Figure 2:**
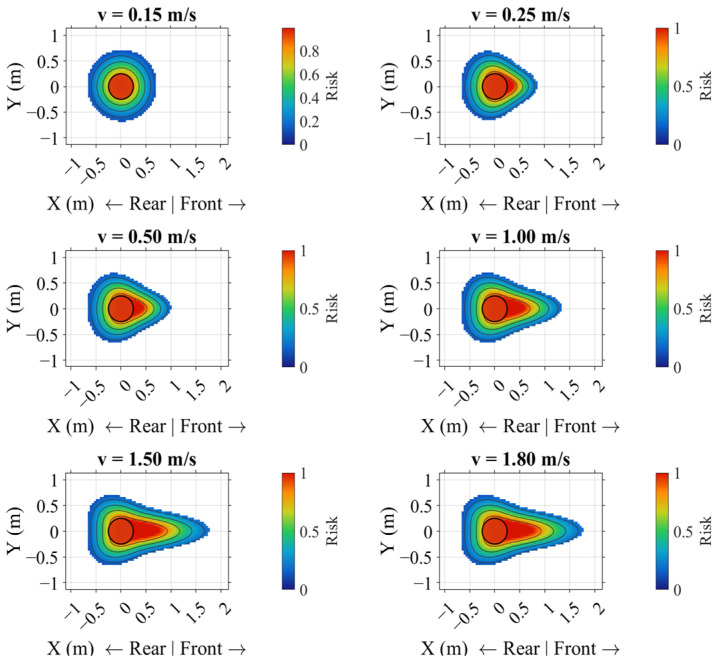
Heatmaps of the risk field at different velocities. The “Front” and “Rear” correspond to the moving direction of the dynamic obstacles. The innermost bold circle represents the actual size of the obstacle, while the outer lines delineate the boundaries of different risk levels, with the risk value decreasing gradually from the center outwards.

As depicted in Figure 2, the risk field maintains an isotropic circular profile when the velocity v is strictly less than the defined threshold $v_{threshold}=0.25$ m/s. Once v reaches or exceeds this limit, the algorithm initiates the frontal elongation logic described in Algorithm 2; consequently, at 0.25 m/s, the field begins to exhibit initial anisotropic elongation, marking the transition from a quasi-static to a velocity-dependent state. Beyond this limit, the field elongates proportionally along the motion direction and is strictly capped to prevent unbounded expansion. By explicitly modeling velocity, this anisotropic mechanism transcends static approximations, enabling the planner to actively steer paths behind or alongside dynamic obstacles. This effectively avoids head-on collisions from aggressive front-cutting and eliminates controller oscillations induced by routing between converging obstacles, ensuring robust navigation in complex environments.

### 3.3. Spatiotemporal Trajectory Post-Processing

Although the initial path is safe and navigable, RRT’s inherent limitations inevitably yield tortuous, zigzagging trajectories. This phenomenon introduces redundant nodes and unnecessarily inflates the overall path length.

#### 3.3.1. Line-of-Sight Path Simplification

The Line-of-Sight algorithm is a classic post-processing technique utilized to prune redundant intermediate nodes from initial zigzagging RRT trajectories. However, traversing this shortened path at maximum velocity may violate the original safety time windows, causing premature arrivals at conflict zones and subsequent collisions with dynamic obstacles. To address this, we introduce a “waiting strategy” at designated safe nodes to reallocate path timestamps. This mechanism geometrically compresses the trajectory while strictly preserving the original safety windows, completely averting collision risks induced by premature advancement.

#### 3.3.2. Bézier Curve Smoothing

**Figure 3:**
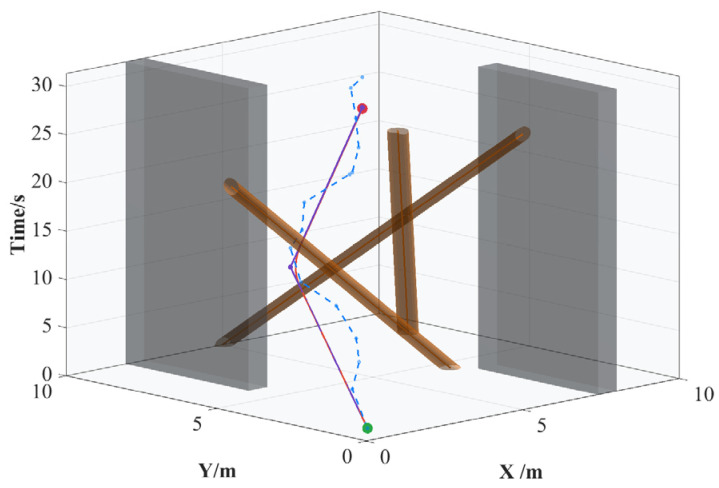
The path post-processing procedure. The blue dashed line represents the original path, the purple line denotes the LOS-simplified path, and the red line indicates the Bézier-smoothed path. Additionally, the green and red dots represent the start and end positions, respectively.

We employ piecewise local Bézier curve optimization over computationally intensive B-splines to ensure both algorithmic efficiency and trajectory safety. During optimization, the algorithm first isolates and preserves in-place waiting events, maintaining the original collision avoidance logic. Sharp corners are then iteratively smoothed using descending smoothing distances $d_{smooth}$. Crucially, this refinement operates exclusively within the 2D spatial domain (x, y), keeping the time dimension t unaltered to prevent timestamp distortion. To guarantee absolute safety, a smoothed arc is retained only if it strictly satisfies both static obstacle and dynamic risk field constraints. The final trajectory, refined via spatiotemporal LOS pruning and Bézier smoothing, is illustrated in Figure 3.

As illustrated in Figure 3, the LOS-pruned trajectory (purple segments) significantly compresses the total path length and concurrently minimizes temporal costs. Furthermore, the Bézier-smoothed path (red trajectory) enforces strict curvature continuity. Ultimately, this dual-stage refinement equips the robot with both high navigational efficiency and kinematic stability across diverse scenarios.

## 4. Ablation Studies and ROS2 Simulation Analysis

### 4.1. MATLAB-Based Ablation Studies

To validate the computational efficiency of the proposed optimizations in dynamic environments, an ablation study was conducted in MATLAB R2024b (MathWorks, Natick, MA, USA) across three scenarios with varying dynamic obstacle densities. The four evaluated variants are configured as follows:

Algorithm A: Standard RRT with an isotropic circular Gaussian risk field;

Algorithm B: Three-layer probabilistic hybrid RRT with an isotropic circular Gaussian risk field;

Algorithm C: Standard RRT with the proposed TRF;

Algorithm D: Three-layer probabilistic hybrid RRT with the proposed TRF.

#### 4.1.1. Map Construction

**Figure 4:**
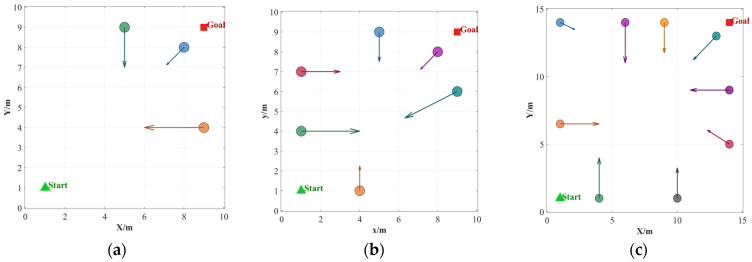
Dynamic obstacle maps: (**a**) Map-a: Three dynamic obstacles; (**b**) Map-b: Six dynamic obstacles; (**c**) Map-c: Nine dynamic obstacles. The differently colored dots represent the starting positions of various dynamic obstacles, and the corresponding arrows indicate their moving directions.

As depicted in Figure 4, three test scenarios featuring varying dynamic obstacle densities were configured within MATLAB 2024b:

The motion vectors of all dynamic obstacles are depicted in Figure 4. To streamline the presentation, the kinematic profiles of the obstacles are formalized as tuples (Start Coordinate, Goal Coordinate, Velocity). The specific configurations for the three test environments are detailed below:

The kinematics data of all dynamic obstacles in the map are detailed in Table 1.

#### 4.1.2. Choice of Sampling Proportions

To validate the rationality and environmental generalizability of the probability distribution in the three-layer hybrid sampling strategy, a parameter sensitivity analysis was conducted on three distinct configurations within maps incorporating the teardrop-shaped risk field. All results were averaged over 30 independent trials. The specific sampling proportions are defined as follows:

Proportion 1: 25% goal-biased, 40% A*-guided heuristic, and 35% uniform random sampling. This proportion is designed to achieve the optimal balance between global directional pull and local dynamic evasion.

Proportion 2: 30% goal-biased, 35% A*-guided heuristic, and 35% uniform random sampling. By increasing the goal bias weight, this proportion drives the algorithm to explore greedily toward the target.

Proportion 3: 20% goal-biased, 45% A*-guided heuristic, and 35% uniform random sampling. By intensifying the heuristic guidance, this proportion forces the planner to rely strictly on safe corridors for conservative navigation.

**Figure 5:**
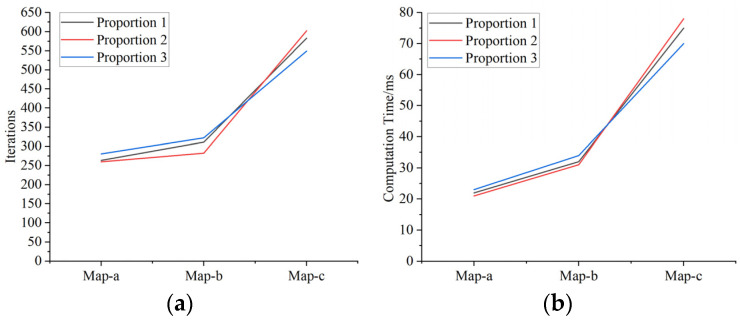
Sensitivity Analysis: (**a**) Iterations; (**b**) Computation Time.

As illustrated in Figure 5, the average results of 30 independent trials across the three maps indicate that the proposed probability distribution does not necessarily yield the absolute minimum computation time and number of iterations in every individual map. However, it maintains exceptional stability and balance across all environments. It successfully avoids the drastic performance degradation exhibited by other extreme configurations in specific scenarios, thereby achieving the optimal global generalizability.

#### 4.1.3. Comparative Analysis of Ablation Studies

Both Map-a and Map-b have dimensions of 10 m × 10 m, with the start point located at (1, 1) and the goal point at (9, 9). Meanwhile, Map-c has dimensions of 15 m × 15 m, with the start point at (1, 1) and the goal point at (14, 14). The relevant experimental parameters are summarized in Table 2:

As detailed in Table 2, the robot is assigned a 0.15 m bounding radius to represent a typical indoor differential-drive platform. To account for this physical footprint within our algorithmic framework, we apply configuration space inflation, whereby the base radius of the TRF is radially expanded by this exact safety margin. Naturally, assuming a larger robot radius would correspondingly expand both the obstacle footprints and the overall TRF boundaries. While this guarantees absolute safety, it compels the planner to trigger avoidance behaviors earlier, yielding longer detour paths and potentially degrading the success rate of finding feasible trajectories in narrow, highly congested dynamic environments.

**Figure 6:**
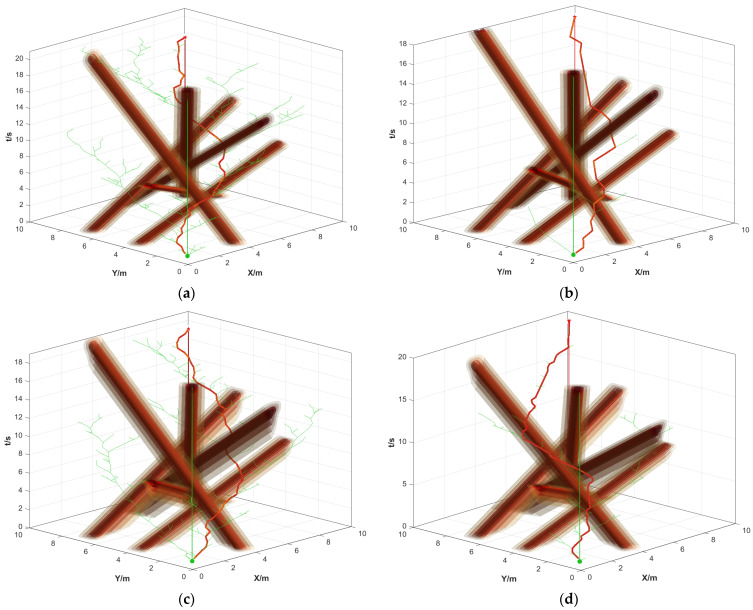
Ablation study comparison in Map-b: (**a**) Algorithm A; (**b**) Algorithm B; (**c**) Algorithm C; (**d**) Algorithm D. The green circle and red star denote the start and end spatiotemporal positions. Cyan segments represent the RRT tree, and red lines show the original paths.

Table 3 summarizes the average computation time and number of iterations for the four ablation algorithms across 30 independent trials. In conjunction with Figure 6, these results illustrate the performance evolution of the spatiotemporal RRT framework across environments of varying complexity (Map-a to Map-c), thereby validating the proposed module.

In the most complex scenario, Map-c, compared with Algorithm A, the proposed Algorithm D reduced the average number of iterations from 949.5 to 538.1—a reduction of 43.33%—and shortened the computation time from 211 milliseconds to 75 milliseconds, a reduction of 64.45%. This significant acceleration stems from two mechanisms: (1) Heuristic sampling limits blind exploration within the high-dimensional spatiotemporal space; (2) the risk field pre-prunes high-risk regions, greatly reducing the frequency of collision detection calls.

Conversely, a comparison between Algorithm B and Algorithm D reveals the computational trade-offs introduced by TRF. In Map-c, TRF enforces stricter safety margins by significantly compressing the navigable free space. These stringent spatial constraints introduce additional overhead, resulting in a 102.7% increase in computation time and a 120.99% increase in the number of iterations compared to the isotropic baseline.

### 4.2. System-Level Validation via ROS2 Simulations

To transcend the limitations of idealized simulations, high-fidelity 3D environments were constructed in Gazebo. These scenarios replicate the start and goal configurations of Figure 4, augmented with sparse static obstacles. By deploying agents with rigorous collision meshes and physical dynamics, this phase evaluates the algorithm’s robustness against sensor blind spots and its strict compliance with real-world kinematic constraints. The hardware and software specifications for these simulations are detailed in Table 4:

The robot’s specific parameters are summarized in Table 5:

#### 4.2.1. Experimental Results and Analysis

To comprehensively benchmark performance in complex dynamic environments, the ROS2 Humble (Open Robotics, Mountain View, CA, USA) simulations compare the proposed approach against three established baselines: ORCA, MPPI, and ST-RRT*. These algorithms strategically represent three distinct planning paradigms: myopic local reaction, finite-horizon predictive dynamics, and global asymptotic spatiotemporal search, respectively.

**Figure 7:**
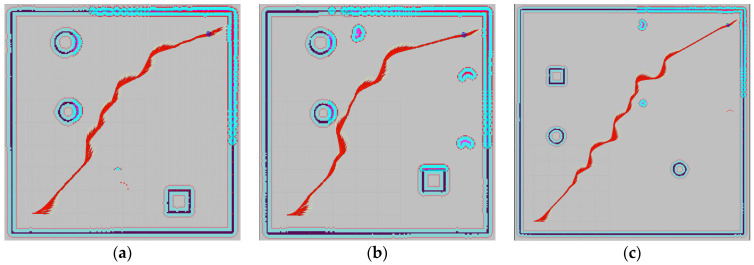
Driving trajectories of the ORCA algorithm in RViz: (**a**) Trajectory in Map-a; (**b**) Trajectory in Map-b; (**c**) Trajectory in Map-c. Red paths with arrows represent robot trajectories, and black areas denote static obstacles. The dimly and brightly highlighted regions surrounding the obstacles indicate the global and local inflation layers, respectively.

**Figure 8:**
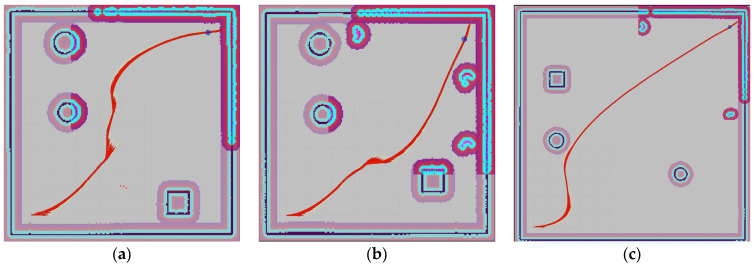
Driving trajectories of the MPPI algorithm in RViz: (**a**) Trajectory in Map-a; (**b**) Trajectory in Map-b; (**c**) Trajectory in Map-c. The meanings of the colors and shapes are the same as those in Figure 7.

**Figure 9:**
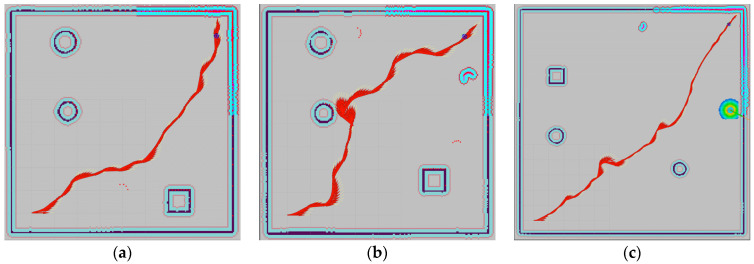
Driving trajectories of the ST-RRT* algorithm in RViz: (**a**) Trajectory in Map-a; (**b**) Trajectory in Map-b; (**c**) Trajectory in Map-c. The heatmap represents the dynamic obstacle risk field, and the meanings of the remaining colors and shapes are the same as those in Figure 7.

**Figure 10:**
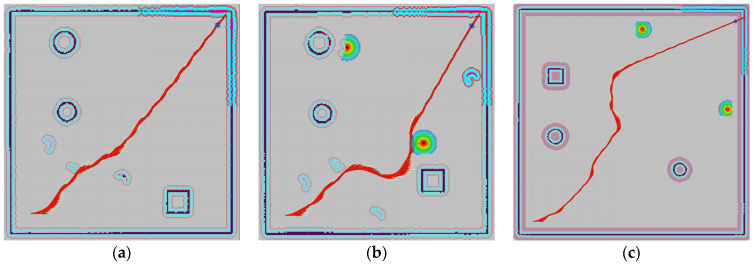
Driving trajectories of the TRF-ST-RRT algorithm in RViz: (**a**) Trajectory in Map-a; (**b**) Trajectory in Map-b; (**c**) Trajectory in Map-c. The heatmap represents the dynamic obstacle risk field, and the meanings of the remaining colors and shapes are the same as those in Figure 7.

To mitigate stochastic variance and rigorously assess algorithmic robustness, 30 independent navigation trials were executed for each method. Overall success rates were aggregated, and key performance metrics for successful runs—specifically, total planning time, total travel time, and trajectory distance—are quantitatively summarized in Table 6:

The trajectory results shown in Figure 7, Figure 8, Figure 9 and Figure 10 demonstrate significant differences in obstacle avoidance behavior among the various evaluation algorithms. Due to the lack of global guidance, ORCA relies entirely on reactive maneuvers, resulting in abrupt detours and severe jitter, which violates kinematic smoothness. Although MPPI improves trajectory smoothness, its limited optimization time window often leads to overly conservative maneuvers, thereby producing sub-optimal deviations in multi-obstacle scenarios. The classical ST-RRT* suffers from control degradation in highly dynamic environments; as it is unable to maintain a sufficient forward safety buffer, the new path generated after replanning may force the robot to make sharp turns to avoid obstacles. Furthermore, even during obstacle-free phases, the dual requirements of reconnecting to parent nodes and strictly tracking arrival times trigger continuous steering corrections, thereby compromising the smoothness of navigation.

As shown in Table 6, compared with the local planning algorithms ORCA and MPPI, joint spatiotemporal search is capable of proactively avoiding dynamic conflicts, thereby effectively resolving the issue of a narrow field of view. In contrast, the proposed TRF-ST-RRT framework, through the integrated application of LOS pruning, Bézier smoothing and joint spatiotemporal planning, effectively mitigates the risks of inefficiency and oscillatory instability, maintaining continuous and efficient trajectories in complex environments. This method achieves significant improvements over the global progressive optimal algorithm ST-RRT*. Firstly, the three-layer probabilistic mixed sampling mechanism significantly reduces computational overhead, with the Map-c metric reduced by 81.75%, thereby ensuring real-time driving performance. Secondly, TRF effectively addresses the critical weakness of insufficient safety margins during head-on dynamic encounters. By actively extending the forward boundary, TRF effectively compensates for the inevitable system delays caused by state replanning, tree structure reconstruction and kinematic steering, resulting in a 13.33%, 16.67% and 10% improvement in the Map-c obstacle avoidance success rate compared to ORCA, MPPI and ST-RRT*, respectively. Finally, the synergy between this hybrid sampling mechanism and TRF ensures robust responsiveness and high success rates in highly congested dynamic environments. Meanwhile, LOS time reallocation improves Map-c’s driving efficiency by 6.7%, 21.1% and 19.8% compared to ORCA, MPPI and ST-RRT* respectively, while consistently guaranteeing kinematic efficiency across all evaluation scenarios.

Nevertheless, as shown in Table 6 and Figure 7, Figure 8, Figure 9 and Figure 10, the algorithm proposed in this paper achieves the highest success rate and driving efficiency, and its advantages demonstrate excellent scalability as environmental complexity increases. The algorithm’s planning time is relatively long in complex maps, which is an inherent characteristic of high-dimensional spatiotemporal search. Furthermore, trade-offs exist in terms of path optimization; for example, in Map-c, the planner adopts a more circuitous route to maintain sufficient dynamic safety margins, thereby fundamentally prioritizing absolute navigation safety in dense, obstacle-rich environments.

**Figure 11:**
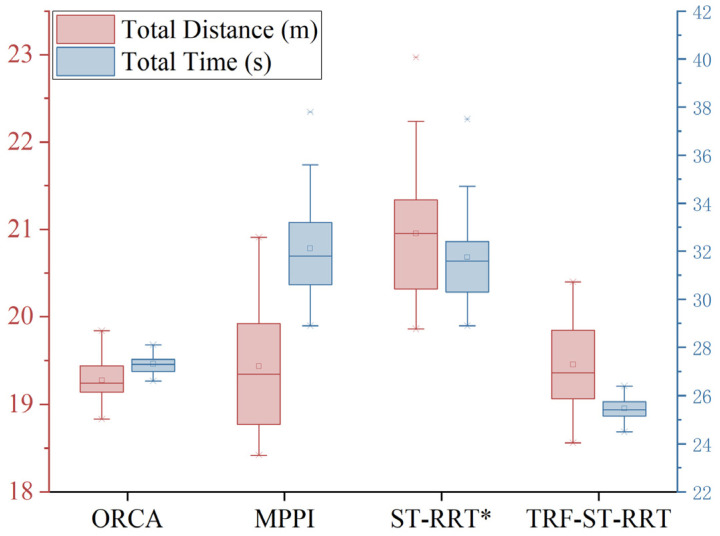
Dual-axis box plot of total distance and total time for different algorithms. In each box, the horizontal line represents the median, the small square denotes the mean, the “x” mark indicates outliers, and the upper and lower ends of the whiskers represent the maximum and minimum non-outlier values.

As shown in Figure 11, the dual-axis box plot illustrates the performance distribution of the four algorithms regarding total navigation distance and total driving time. Regarding time efficiency, the TRF-ST-RRT algorithm consistently achieves the lowest latency and exhibits minimal variance, demonstrating superior operational stability compared to the pronounced delays and significant fluctuations observed in MPPI and ST-RRT*. In terms of path efficiency, ORCA generates the shortest trajectories due to its greedy local-reactive nature, whereas TRF-ST-RRT maintains a highly competitive trajectory length that closely matches ORCA while significantly outperforming the global search strategy of ST-RRT*. Although TRF-ST-RRT displays slight variations in path distance, this is a deliberate trade-off to maintain sufficient safety buffers around dynamic obstacles, confirming the algorithm’s capability to effectively balance navigational efficiency, kinematic stability, and safety.

#### 4.2.2. Kinematic Performance and Obstacle Avoidance Analysis

**Figure 12:**
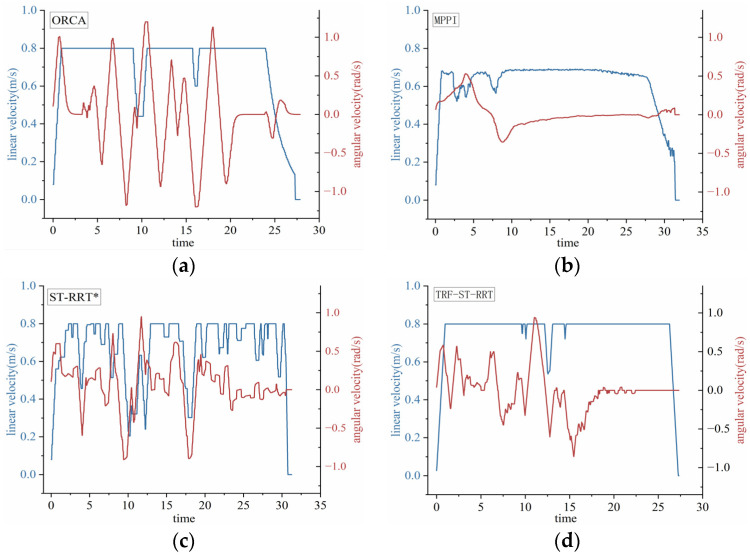
Linear and angular velocity profiles: (**a**) ORCA; (**b**) MPPI; (**c**) ST-RRT*; (**d**) TRF-ST-RRT.

Figure 12 delineates the distinct kinematic discrepancies among the evaluated algorithms in Map-c. First, ORCA’s myopic nature triggers abrupt emergency braking and induces sinusoidal angular oscillations even on obstacle-free straightaways, which subsequently couple into linear velocity fluctuations. Second, MPPI exhibits pronounced control noise; its finite optimization horizon disrupts trajectory continuity, while Monte Carlo stochasticity injects persistent high-frequency chatter into the linear velocity, degrading transport efficiency. Third, conventional ST-RRT* suffers from severe kinematic instability; enforcing strict pre-planned arrival schedules compels the tracking controller to aggressively correct toward newly rewired trajectories, yielding erratic control inputs.

Conversely, by sequentially integrating LOS pruning and Bézier smoothing within the joint spatiotemporal architecture, the proposed framework preserves exceptional kinematic smoothness. Except during large-angle evasive maneuvers against proximate dynamic obstacles, the robot sustains its maximum linear velocity, substantially optimizing operational efficiency.

However, as a necessary trade-off for absolute safety, it must be acknowledged that while each individually generated trajectory is spatially smooth, the proposed TRF-ST-RRT algorithm inevitably exhibits a certain degree of heading oscillation during navigation, as depicted in Figure 12d. This phenomenon stems primarily from the highly reactive nature of the planner. Because the path is continuously replanned in real time to accommodate the updated positions, velocities, and moving directions of dynamic obstacles, the robot is forced to frequently adjust its heading to strictly track these dynamically shifting trajectories. This effect is further amplified in scenarios with physical occlusions. When an obstacle suddenly becomes visible, the planner must immediately enforce a spatial safety margin equivalent to the dynamic TRF boundary. Guaranteeing this strict collision-free clearance often necessitates sharp, substantial steering maneuvers to proactively evade the threat.

**Figure 13:**
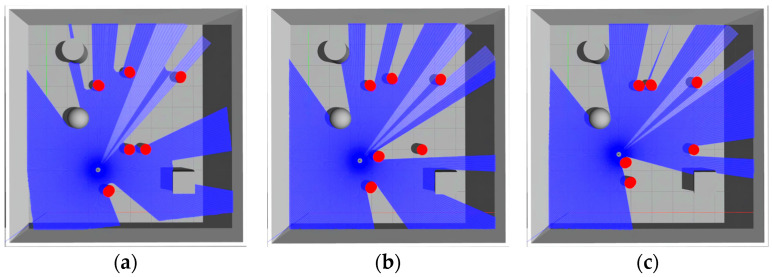
The process of lateral obstacle avoidance: (**a**) Before avoidance; (**b**) During avoidance; (**c**) After avoidance. In the figure, blue lines represent Lidar beams, red objects are dynamic obstacles, and gray shaded areas denote static obstacles. The gray object at the center of the Lidar beams is the robot.

**Figure 14:**
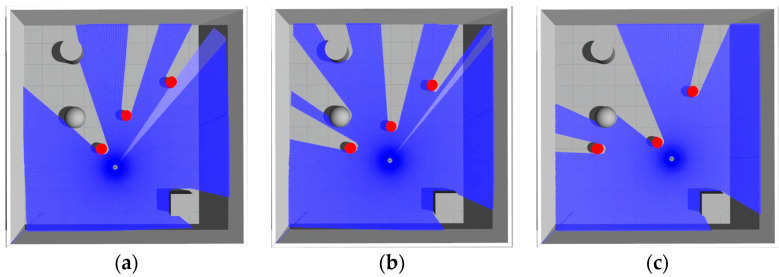
The process of frontal obstacle avoidance: (**a**) Before avoidance; (**b**) During avoidance; (**c**) After avoidance. The meanings of the colors and shapes are the same as those in Figure 13.

As illustrated in Figure 13 and Figure 14, the sequential stages (before, during, and after) of lateral and frontal avoidance highlight the robot’s lateral evasive maneuvers and spatiotemporal yielding behaviors when confronting dynamic obstacles. The experimental observations reveal that the robot consistently maintains the predefined safety margin. This performance directly validates the efficacy of the proposed risk field. Specifically, the risk field actively compels the planner to secure an adequate safe distance ahead of the moving obstacle’s trajectory. Consequently, this mechanism mitigates the spatial compression induced by frontal threats, fundamentally preventing physical collision deadlocks typically triggered by computational latency during replanning.

### 4.3. Analysis of Failure Cases

**Figure 15:**
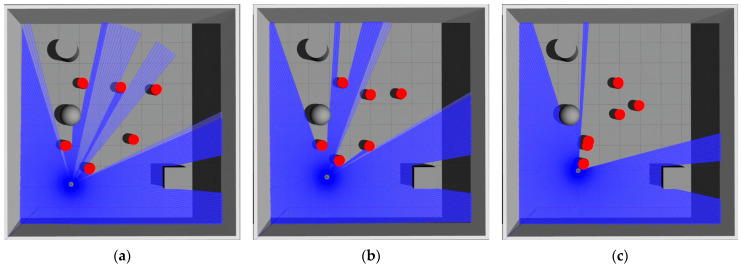
Physical occlusion failure process: (**a**) The obstruction is about to end; (**b**) During avoidance; (**c**) The collision occurred. The meanings of the colors and shapes are the same as those in Figure 13.

In a total of 90 independent ROS2 evaluation trials, as anticipated, the traditional planner achieved an unsatisfactory obstacle avoidance success rate in complex environments when encountering high-speed dynamic obstacles, due to issues such as short-sightedness and lock-in. The algorithm proposed in this paper maintained a high navigation success rate, reaching 93.33% in both Map-b and Map-c; the specific statistical results are shown in Table 6. The few instances of failure were primarily attributable to three interrelated bottlenecks. Firstly, sensor perception errors can severely degrade performance, as joint spatiotemporal planning fundamentally relies on precise estimates of an obstacle’s velocity, heading, position and geometry. When significant random noise occurs in LiDAR ranging or velocity tracking, the cumulative deviations in the predicted trajectory inevitably lead to inaccuracies in collision risk assessment. Secondly, as shown in Figure 15, perception blind spots caused by single-sensor occlusion pose a major challenge; dynamic obstacles are frequently obscured by static structures or surrounding objects. When an occluded target suddenly re-enters the field of view, robots constrained by rigid kinematic limitations lack sufficient temporal and spatial buffer to evade rapidly approaching threats. Thirdly, in ultra-dense environments, the TRF mechanism exhibits an inherent trade-off; the aggregation of strictly enforced safety buffers can excessively constrain the navigable free space, occasionally resulting in a “lock-in” phenomenon where the planner fails to identify a feasible trajectory. While adaptive adjustment of the risk field radius could potentially mitigate this, such an approach requires a careful balance against the necessity of maintaining sufficient safety clearance. Furthermore, ST-RRT* does not reserve sufficient safety margins to accommodate replanning and transition to new paths, resulting in a lower obstacle avoidance success rate compared to TRF-ST-RRT. To further improve the success rate of the spatiotemporal framework, future research will focus on multimodal sensor fusion and high-fidelity object tracking. Integrating complementary modalities, such as cameras and millimeter-wave radar, will significantly enhance perception capabilities within occluded regions and improve the accuracy of obstacle state estimation.

Finally, this experiment was primarily conducted in an environment where the vehicle traveled in a straight line at a constant speed amidst ideal dynamic obstacles; the construction of the current risk field relies on linear kinematic predictions. In future research, we will introduce models such as pedestrian trajectory prediction to address non-linear, highly random dynamic obstacles.

## 5. Conclusions

To address the challenge of balancing computational efficiency and safety in dynamic spatiotemporal path planning, this paper proposes the TRF-ST-RRT algorithm. The main contributions and conclusions are summarized as follows:

(1) A teardrop risk field based on velocity vectors: The core innovation is the velocity-dependent TRF model, which transitions from traditional isotropic Gaussian fields to an anisotropic structure that dynamically scales frontal safety corridors according to emergency braking requirements. This improvement resolves the inherent conflict between excessive lateral conservatism and inadequate frontal collision margins. To ensure the real-time deployment of this field in high-dimensional spatiotemporal searches, we integrated a three-layer probabilistic hybrid sampling strategy. This supporting mechanism—comprising goal-biased, A*-heuristic, and uniform exploration—significantly alleviates computational bottlenecks and accelerates tree growth while rigorously preserving the framework’s probabilistic completeness.
(2) LOS path simplification and segmented Bézier curve smoothing: Following two stages of post-processing, driving efficiency and ride comfort were effectively improved, with formal efficiency gains of 6.7%, 21.1% and 19.8% compared to ORCA, MPPI and ST-RRT*, respectively.

## Figures and Tables

**Table 1:** Dynamic obstacle data.

Map	Dynamic Obstacle ID	Start Point	Goal Point	Linear Velocity (m/s)	Map	Dynamic Obstacle ID	Start Point	Goal Point	Linear Velocity (m/s)
a	1	(9, 5)	(1, 5)	1.2	c	1	(1, 6)	(14, 6)	1.2
	2	(8, 8)	(1, 1)	0.5		2	(4, 1)	(14, 1)	1.2
	3	(5, 9)	(5, 1)	0.8		3	(1, 14)	(14, 8)	0.8
b	1	(4, 1)	(4, 9)	0.5		4	(14, 5)	(1, 13)	0.5
	2	(1, 4)	(9, 4)	1.2		5	(6, 14)	(6, 1)	1.2
	3	(5, 9)	(5, 1)	0.6		6	(9, 14)	(9, 1)	0.8
	4	(1, 7)	(9, 7)	0.8		7	(13, 13)	(1, 1)	0.8
	5	(8, 8)	(1, 1)	0.5		8	(14, 9)	(1, 9)	1.2
	6	(9, 6)	(1, 2)	1.2		9	(10, 1)	(10, 13)	0.8

**Table 2:** Robot-related parameters used in the simulation experiments.

Parameter	Value
Robot radius	0.15 m
Dynamic obstacle radius	0.25 m
Safety threshold	0.45 m
Maximum robot velocity	0.8 m/s
Maximum obstacle velocity	1.5 m/s
Discrete-time safety factor α	0.5
Time step $\;∆t_{opt}$	0.14 s
Goal bias probability	25%
Heuristic-guided search probability	40%
Random search probability	35%
Risk decay coefficient	0.3
Base safety radius of the risk field	0.45 m
Maximum frontal extension of the risk field	1.5 m

**Table 3:** Comparative results of the ablation studies.

Algorithm	Map	Iterations	Computation Time (ms)
A	a	441	44
	b	445.9	66
	c	949.5	211
B	a	130.3	15
	b	144.9	19
	c	243.5	37
C	a	486.5	54
	b	508.7	89
	c	984.6	241
D	a	263.4	22
	b	311.2	32
	c	538.1	75

**Table 4:** System specifications of the experiments.

Component	Specification
CPU	Intel^®^ Core™ i7-14700HX
GPU	NVIDIA GeForce RTX4070 Laptop GPU 8 GB
RAM	16 GB, DDR5, 5600 Hz
Operating system	Ubuntu 22.04
ROS2 version	Humble

**Table 5:** Parameters of the experimental robot.

Experimental robot radius	0.15 m
Maximum linear velocity	0.8 m/s
Acceleration	0.8 m/s^2^
Maximum angular velocity	1.2 rad/s
Maximum LiDAR detection range	8 m

**Table 6:** Comparative performance results.

Algorithm	Map	Total Time (s)	Total Planning Time (ms)	Total Distance (m)	Success Rate (%)
ORCA	a	17.65 ± 0.3	2.97 ± 0.25	11.92 ± 0.13	100
	b	18.14 ± 0.31	3.16 ± 0.16	12.36 ± 0.14	86.67
	c	27.29 ± 0.4	4.75 ± 0.36	19.23 ± 0.24	80
MPPI	a	20.01 ± 0.5	1715.85 ± 90.93	11.77 ± 0.13	93.33
	b	22.48 ± 0.77	1743.29 ± 214.36	12.97 ± 0.58	83.33
	c	32.25 ± 2.14	2421.38 ± 246.87	19.42 ± 0.7	76.67
ST-RRT*	a	20.45 ± 1.94	1815.2 ± 130.76	12.77 ± 0.39	100
	b	22.23 ± 2.22	2952.73 ± 429.39	12.91 ± 0.55	90
	c	31.75 ± 1.84	3569.45 ± 454.35	19.95 ± 0.75	83.33
TRF-ST-RRT	a	16.45 ± 0.9	133.06 ± 24.89	12.07 ± 0.27	100
	b	17.38 ± 0.9	145.71 ± 22.28	12.1 ± 0.37	93.33
	c	25.46 ± 0.56	651.35 ± 87.94	19.55 ± 0.49	93.33

## Data Availability

The original contributions presented in the study are included in the article; further inquiries can be directed to the corresponding author.

## Abbreviations

The following abbreviations are used in this manuscript:

ST-RRT*	Spatiotemporal Rapidly exploring Random Tree Star
VO	Velocity Obstacle
ORCA	Optimal Reciprocal Collision Avoidance
MPPI	Model Predictive Path Integral
TRF-ST-RRT	Teardrop Risk Field Spatiotemporal Rapidly exploring Random Tree
TRF	Teardrop Risk Field
LOS	Line of Sight
